# Mesenchymal stem cells overexpressing Ihh promote bone repair

**DOI:** 10.1186/s13018-014-0102-7

**Published:** 2014-10-28

**Authors:** Shasha Zou, Tingting Chen, Yanan Wang, Ruhui Tian, Lingling Zhang, Pingping Song, Shi Yang, Yong Zhu, Xizhi Guo, Yiran Huang, Zheng Li, Lixin Kan, Hongliang Hu

**Affiliations:** Renji Hospital, School of Medicine, Shanghai Jiao Tong University, 845 Lingshan Road, Shanghai, 200135 China; BIO-X Center, Shanghai Jiao Tong University, 55 Guangyuan West Road, Shanghai, 200240 China; Department of Pathophysiology, School of Basic Medicine, Anhui Medical University, 81 Meishan Road, Hefei, Anhui 230032 China; Feinberg School of Medicine, Northwestern University, 303 East Chicago Avenue, Chicago, IL 60611 USA

**Keywords:** Indian hedgehog (Ihh), Chondrogenesis, Osteogenesis, Angiogenesis, Mesenchymal stem cells (MSCs), Tissue engineering

## Abstract

**Background:**

Indian hedgehog (Ihh) signaling pathway is known to play key roles in various aspects of normal endochondral bone development. This study tested the potential roles of high Ihh signaling in the context of injury-induced bone regeneration.

**Methods:**

A rabbit tibia defect model was established to test the effects of the implant of Ihh/mesenchymal stem cells (MSCs)/scaffold complex. Computed tomography (CT), gross observation, and standard histological and immunohistological techniques were used to evaluate the effectiveness of the treatment. *In vitro* studies with MSCs and C3H10T1/2 cells were also employed to further understand the cellular and molecular mechanisms.

**Results:**

We found that the implanted Ihh/MSCs/scaffold complex promoted bone repair. Consistently, *in vitro* study found that Ihh induced the upregulation of chondrocytic, osteogenic, and vascular cell markers, both in C3H10T1/2 cells and MSCs.

**Conclusions:**

Our study has demonstrated that high Ihh signaling in a complex with MSCs enhanced bone regeneration effectively in a clinically relevant acute injury model. Even though the exact underlying mechanisms are still far from clear, our primary data suggested that enhanced chondrogenesis, osteogenesis, and angiogenesis of MSCs at least partially contribute to the process. This study not only has implications for basic research of MSCs and Ihh signaling pathway but also points to the possibility of direct application of this specific paradigm to clinical bone repair.

**Electronic supplementary material:**

The online version of this article (doi:10.1186/s13018-014-0102-7) contains supplementary material, which is available to authorized users.

## Introduction

Bone graft, such as autogenous iliac crest bone graft (ICBG), is currently the most common approach for bone defect repair [1], largely because of its impressive osteoinductive, osteogenic, and osteoconductive abilities [2,3]. However, this approach causes high morbidity (~31%) of the iliac crest bone donors [4], which has significantly limited its application in clinical settings. Therefore, alternative approaches with the tissue-engineered bone substitutes have been widely investigated. However, many common problems, such as the insufficient blood supply of the tissue engineering bone [5] and the lack of osteoinduction or osteogenesis capacity of polymeric scaffolds [6] still have not been adequately addressed.

Local application of certain factors, such as bone morphogenetic proteins (BMPs) [7], fibroblast growth factors (FGFs) [8], vascular endothelial growth factors (VEGFs), RUNX2 in different contexts [9,10], and other bioactive factors [11], and even systematic administration of antibody against sclerostin [12], have been proved to be beneficial for bone repair. However, the effects of local application of Indian hedgehog (Ihh) have not been systematically tested, even though it has been recently reported that the activation of hedgehog signaling during fracture repair enhanced osteoblastic-dependent matrix formation [13].

Ihh, a member of hedgehog (Hh) family, has been investigated largely as an important regulatory factor in normal embryonic and neonatal bone development. In this context, Ihh is thought to be a master regulator of both chondrocyte and osteoblast differentiation, working largely through regulatory feedback loops. In addition, Ihh was also implicated in angiogenesis and vascularization of newly formed bone [14,15].

Ihh receptors, both patched (Ptc,a conserved 12-transmembrane protein) and smoothened (Smo, a 7-transmembrane protein), are conserved. It is known that secreted Hh proteins bind to receptor Ptc. The subsequent downstream signaling cascade, through receptor Smo to the Ci or Gli1–3 transcription factors, can either activate or inhibit specific target genes [16,17].

Furthermore, it is known that endogenous mesenchymal stem/progenitor cells (MSCs) contribute to the maintenance of healthy tissues or as immunomodulatory sentinels. In consistent with this idea, MSCs from different sources with a variety of modifications have also been widely applied in regenerative medicines in different clinical contexts [18,19]. These numerous studies yielded promising yet often seemly conflicting data.

In this study, we specifically hypothesized that, similar to what we have observed in normal embryonic and neonatal bone development, Ihh signaling through exogenous MSCs might play a crucial role in the adult bone remodeling and regeneration as well, likely working through promoting the chondrogenesis, osteogenesis, and angiogenesis of mesenchymal stem/progenitor cells. Current study is designed to directly test this hypothesis *in vivo* and *in vitro*. This study not only has great implications for basic research of Ihh signaling pathway and MSCs but also suggested the possible direct application of this specific paradigm to clinical bone repair.

## Materials and methods

### Animals

Fifty New Zealand white rabbits (8-weeks old, about 1.5–2.0 kg each) were purchased from Shanghai laboratory animal farm (animal quality number: SCXK Shanghai 2007-0008). All animal experiments were approved and performed in an animal core facility at the Shanghai Institute of Planned Parenthood Research (SIPPR).

### Constructs, transfection and retroviral generation

The flag-tagged human Ihh cDNA, a kind gift from Dr. Gang Ma (Bio-X center, Shanghai Jiao Tong University), was subcloned into the HindIII and EcoRV sites of pCIG-enhanced green fluorescence protein (EGFP), which contains also the EGFP, the internal ribosomal entry site (IRES), and the nuclear localization signal (NLS) sequences for EGFP. The pSFG-EGFP-Ihh (Additional file 1: Figure S1A) and pSFG-EGFP retroviral constructs were subcloned in the similar way and confirmed by DNA sequencing, EGFP^+^ Ihh^+^ viruses and EGFP^+^ viruses were packaged in 293GPG cells as previously described [20]. C3H10T1/2 cells and rabbit bone marrow mesenchymal stem cells were infected with viruses at about 80% confluence in medium supplemented with 8 g/L polybrene for 24 h.

### Cell culture

The C3H10T1/2 cells, the murine mesenchymal stem cells line, were purchased from ATCC (Manassas, VA, USA). 293GPG cells were from the Department of Medicine, Washington University Medical School. Bone marrow MSCs were extracted from femurs and tibias of 2-month-old healthy New Zealand white rabbits. Briefly, the femurs and tibias were first flushed with PBS and disinfected with 70% alcohol sprinkling, and then the bone marrow aspirates were obtained in laminar flow hood with a syringe filled with 2-ml MSC culture medium. MSCs were then isolated by centrifuging for 20 min in 1.073 g/ml Ficoll gradients at 1,000 r/min and then seeded at a density of 1 × 10^6^ cells per 10-cm petri dish in MSC culture medium. After 72 h, the medium was changed to remove non-adherent cells. At 90% confluence, the adherent cells were split at a ratio of 1:3 (p1, passage 1). Passage 3 (p3) of MSCs were routinely used in this study.

Chondrogenic differentiation: chondrogenesis was induced in monolayer in chondrogenic media (CDM1). CDM1 is high-glucose Dulbecco’s modified Eagle’s medium (DMEM) supplemented with 100 ng/ml of BMP2 (R&D Systems Inc. Minneapolis, MN, USA), 10 ng/ml of transforming growth factor-β3, 100-nM dexamethasone, 50 μg/ml of L-ascorbic acid 2-phosphate, 100 μg/ml of pyruvate (Sigma-Aldrich, St. Louis, MO, USA), 40 μg/ml of proline (Sigma-Aldrich), and 10 μl/ml of ITS+ (BD Biosciences, San Jose, CA, USA). Three weeks after induction, cultures were divided into two groups, i.e. in group I, the cells were fixed and stained with 1% Alcian Blue to detect matrix deposition of sulfated glycosaminoglycans (GAGs), and in group II, cells were lysed to extract total RNA, which were used to generate cDNA to probe the transcriptional profile of these cells.

Osteogenic differentiation: osteogenesis was induced by osteogenic differentiation media (ODM1), which consists of low-glucose DMEM supplemented with 10% fetal bovine serum (FBS), 100-nM dexamethasone, 10-mM β-glycerophosphate, and 50-μl M L-ascorbic acid 2-phosphate. On day 21 of cultivation in osteogenic medium, cells were fixed and stained either with BCIP/NBT kit to detect alkaline phosphatase (ALP) or 1% alizarin red S to detect calcified extracellular matrix. Some cells were also lysed to extract total RNA, which were used to generate cDNA to probe the transcriptional profile of these cells.

### ALP, Alcian Blue, and alizarin red staining

Cover slides were fixed in 95% alcohol, and ALP staining was performed with BCIP/NBT kits, according to the manufacturers’ protocols (Beyotime Institute of Biotechnology, Jiangsu, China). The production of GAGs was determined by Alcian Blue staining (Sigma-Aldrich, St. Louis, MO, USA), according to standard protocol. Alizarin red staining was performed according to standard protocol. Briefly, slides were stained with the alizarin red solution for 30 s to 5 min and the reaction was observed microscopically. Excess dye was shaken off, and the sections were dehydrated in acetone, 20 dips, and then in acetone-xylene (1:1) solution, 20 dips. Sections were cleaned in xylene and mounted with a synthetic mounting medium.

### Immunostaining

For immunohistochemistry, cells were plated on gelatinized cover slips in 35-mm petri dish. At about 90%–100% confluence, cell on cover slips were washed, fixed, blocked, and then primary antibody was added and incubated for 12 h at 4°C. After the washes, the secondary antibody was added at 37°C for 1 h. The stained sections were examined and imagined under confocal (Leica Microsystems GmbH, Mannheim, Germany) or optical microscopy (Olympus Optical Co. Ltd, Tokyo, Japan).

### 3-(4,5)-dimethylthiahiazo (-z-y1)-3,5-di-phenytetrazoliumromide (MTT) assay

The cells/HA complexes of the three groups were cultured in 800 μl growth medium on 24-well plates. Five percent MTT was added aseptically to each wells and incubated for 4 h. After the incubation, culture medium was removed, and dimethyl sulfoxide was added. The absorbance (OD) was measured at the wavelength of 570 nm, and the data were analyzed with the software package SPSS version 13.0 (SPSS Inc., Chicago, IL, USA).

### Real-time (RT)-PCR

Total RNA were extracted according to manufacturer’s instructions. cDNA was synthesized with GoScript™ Reverse Transcription System (Promega Biotech Co., San Luis Obispo, CA, USA). RT-PCR was carried out in GeneAmp PCR System 9700 (Applied Biosystems, Foster City, CA, USA). Three independent experiments were performed for each sample with β-actin as internal control. Qualitative PCR (qPCR) assay was performed with SYBR Green master mix (Life Technologies Corporation, Carlsbad, California, USA), as recommended (Applied Biosystems, Foster City, CA, USA), with GAPDH as an internal control.

### The seeding of hydroxyapatite scaffolds

Hydroxyapatite (HA) composite material (Yihuajian Science and Trade Co., Ltd, Beijing, China), batch number 100518, serial number 100518 f704, specifications 45 × 45 × 1.8 mm. The HA scaffolds were cut into 3.5 × 3.5 × 1.8 mm pieces for the current study. P3 rabbit MSCs were divided into three groups to be infected by different viruses (either EGFP^+^ Ihh^+^ virus, or EGFP^+^ virus, or no virus control). Infected cells were then harvested, and 1 × 10^7^ infected cells were then seeded into each HA scaffold. Seeded HA scaffolds were subsequently cultured for four more days, with daily growth medium replacement. The growth and stent of cells on HA scaffold’s surface was measured by fluorescence microscope and scanning electron microscopy (SEM; Philips Quanta-200, FEI Company, Netherlands). At least 80% of the available inner surface of HA scaffolds was covered by seeded cells at the time of implantation.

For SEM observation, the cells/HA complexes were fixed in solutions of 2.0% glutaraldehyde and 1.0% osmic acid. Dehydration of the fixed samples was conducted with acetone and isoamyl acetate, and then the dehydrated cells/HA complexes were coated in vacuum before the scanning.

### Rabbit tibia defect model, the implantation and CT analysis

Tibia defect model was established similar to previous reports [21-23]. Briefly, animals were anesthetized first, and then tibia platforms of hind limbs were exposed, and defects were made on tibial plateau with a 5-mm diameter circular drill bit close to the growth plate. The periosteum and the underlying bony tissue were removed sequentially by the drilling, and the depth of defect is about 2.0 mm, which is less than the thickness of the adjacent cortical bone (Additional file 2: Figure S2). Since this model reflects the features of both the tibial plateau defect and the cortical drill hole defect in the proximal tibia, it was called the tibial plateau defect throughout the paper. Forty New Zealand white rabbits (12-weeks old) were randomly divided into two groups, A and B. Rabbits in group A received EGFP^+^ Ihh^+^ MSCs/HA (left side) and EGFP^+^MSCs/HA (right side), and the group B rabbits received MSCs/HA only (left or right side, randomly).

The shape and the size of the tibia defect (the burr hole) and the implant (cells/HA complexes) were optimized based on our primary study, which found that a drill (circle, 5 mm in diameter) was able to produce a more consistent defect, while a cubical scaffold (3.5 × 3.5 × 1.8 mm) could be generated more consistently. In this paradigm, the cubical scaffold (3.5 × 3.5 × 1.8 mm) can be easily fitted into a burr hole (5 mm in diameter × 2 mm), since the diagonal of the square is largely the same as the diameter of the burr hole (4.95 vs 5 mm), and the height of the implant is close to the depth of the burr hole (1.8 vs 2 mm) (see Additional file 2: Figure S2 for details). The scaffold was secured into the defect by suturing the surrounding soft tissue and skin separately after implantation.

After implantation, postoperative activity, diet, survival condition, complications, and wound healing time were recorded daily. For evaluation of bone formation in the defect sites, computed tomography (CT; Siemens PET—CT 120 kV, 45 mA) scan was performed on the rabbit tibia at four different time points, i.e., 2, 4, 8, and 12 weeks after surgery.

### Gross and histological evaluation of bone regeneration

Animals were sacrificed at 4 and 12 weeks after implantation, and the tibia platforms of hind limbs were exposed and photographed for further analysis. Harvested tissue from the bone defect sites were fixed in 4% PFA immediately, decalcified in 10% EDTA, and embedded in OCT. Frozen sections were used for histological and immunohistochemistrical studies, according to standard protocols.

## Results

### Evaluation of the Ihh overexpression in MSCs and C3H10T1/2 cells

**Figure 1:**
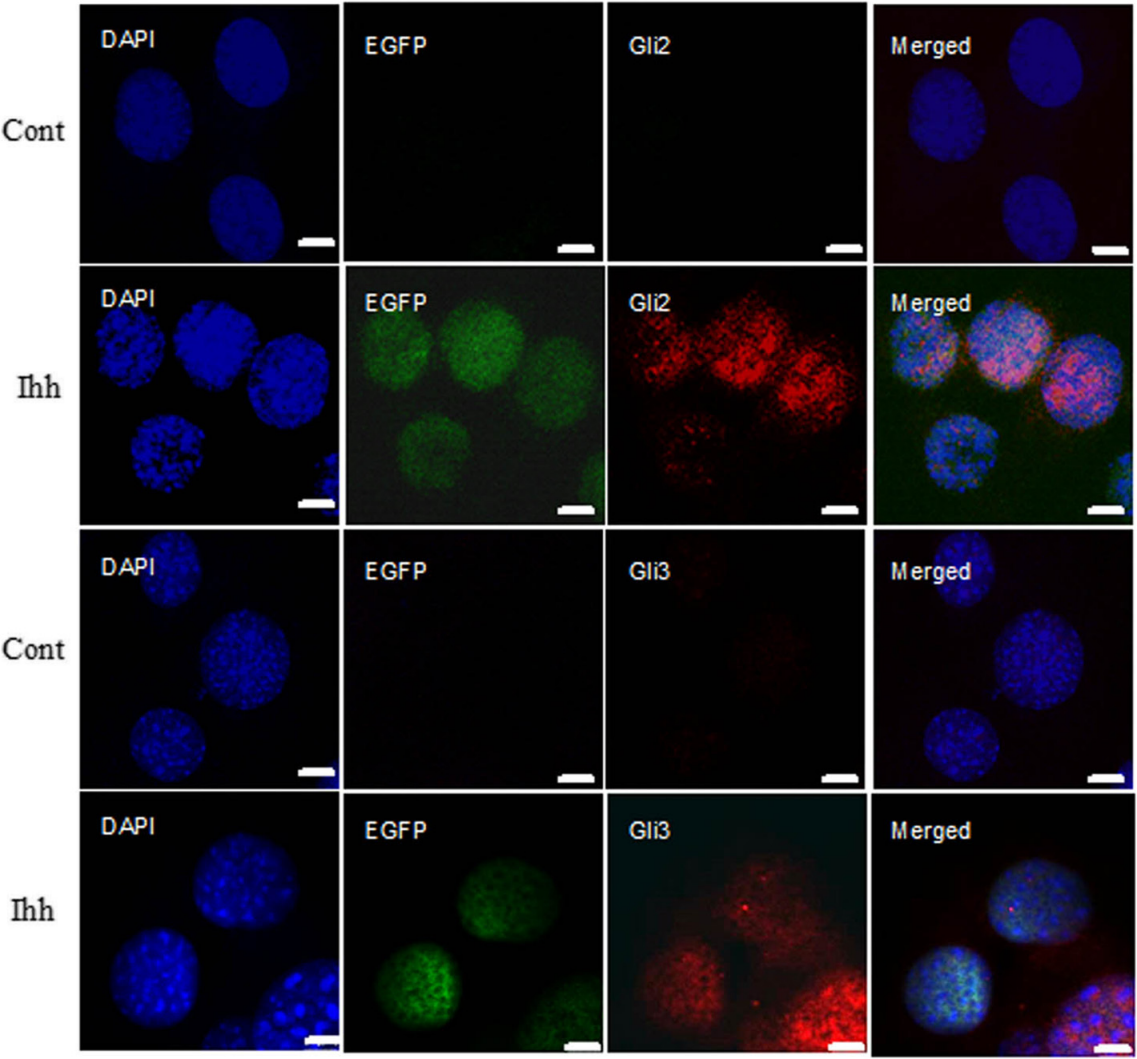
**Overexpression of Ihh in C3H10T1/2 up-regulates Gli2 and Gli3 in the nuclei.** Typical images of double staining demonstrated overexpression of Ihh (GFP^+^, green) co-localized with upregulated Gli2 and Gli3 (red), respectively. The first and third rows were none transfected cells, and the second and fourth rows were transfected cells. The first three columns are the split channels of DAPI (blue), GFP (green), Gli signals (red), respectively, and the fourth column is the merged images of the respective three split channels. Note that the most signals, both Gli2 and Gli3 and GFP, were in the nuclei (DAPI^+^, blue). Scale bars = 5 μm.

We first confirmed that the construct indeed overexpressed Ihh protein in C3H10T1/2 cells, through epifluorescence microscopic imaging and Western blotting (Additional file 1: Figure S1). Furthermore, high level of Hh target genes, Gli2 and Gli3, were detected in the nuclei of EGFP^+^ Ihh^+^ C3H10T1/2 but not in the control cells (Figure 1), strongly suggesting that Ihh overexpression construct worked as we expected.

We next examined the effects of overexpression of Ihh in MSCs and C3H10T1/2 cells, using retrovirus vector pSFG, which usually has higher and more consistent infection efficiency than plasmid construct. The isolated MSCs contained various cells from the bone marrow, but majority of the attached cells are long, slender, and spindle-like shape after splitting (data not shown).

### Evaluation of survival and proliferation of MSCs in HA scaffolds

To directly test whether Ihh overexpressing MSCs can proliferate and survive in HA scaffolds *in vitro*, a prerequisite for the subsequent implantation, we seeded the viral (EGFP^+^ or EGFP^+^ Ihh^+^) infected or control MSCs into HA scaffolds. Two days after seeding, healthy cells were observed both on the outer and internal surfaces of scaffolds in all three groups. On day 7, the surface of HA scaffolds are almost completely covered with tightly arrayed GFP^+^ cells (Additional file 3: Figure S3A), and more importantly, no obvious difference of cell density was observed in different groups (data not shown). SEM further confirmed that cells with a typical spindle-like shape were attached to HA scaffold (Additional file 3: Figure S3B).

To directly monitor the cell viability on scaffolds, MTT assay was performed. Table 1 summarized the viable cell in different groups at different time points. These data indicate that overexpression of Ihh in MSCs did not obviously change the cell proliferation or cell survival on the surface of HA scaffolds.

**Table 1:** **The effects of EGFP or Ihh gene on the proliferation of BMSCs in HA**

	**Time**			
	**Day 1**	**Day 2**	**Day 3**	**Day 4**
BMSCs-HA	0.223 ± 0.007	0.271 ± 0.005	0.352 ± 0.041	0.574 ± 0.017
EGFP^+^ BMSCs-HA	0.223 ± 0.013	0.267 ± 0.016	0.353 ± 0.011	0.573 ± 0.043
EGFP^+^Ihh^+^ BMSCs-HA	0.225 ± 0.009	0.254 ± 0.012	0.310 ± 0.029	0.447 ± 0.021

MTT results presented as OD values of three independent experiments.

### Establishment and evaluation of rabbit tibia defect model

Numerous bone defect models in different species with different defect size and on different sites have been reported in literatures [21]. We established a tibia defect model in rabbit in our current study similar to previous reports [22,23], mainly because not only tibial defect remains one of the major bone defects in clinic but also this model is relatively easy and straightforward with high repeatability. The shape and the size of the defect (the burr hole) and the implant (cells/HA complexes) were optimized based on our primary study (see Additional file 2: Figure S2 and ‘Materials and methods’ section for details) to minimize the intra- and inter-group variations.

**Figure 2:**
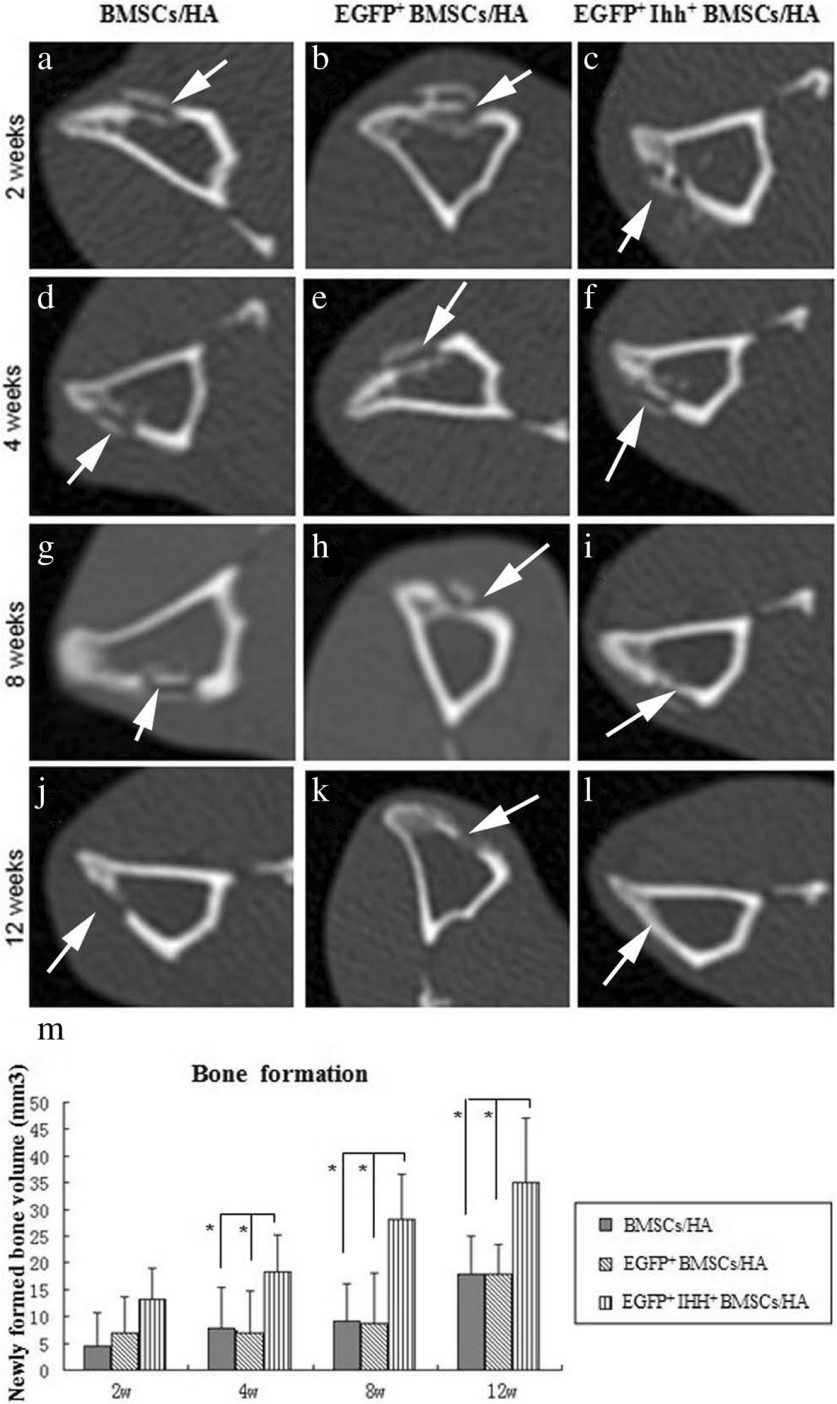
**CT images demonstrated that Ihh** ^**+**^ **MSCs/hydroxyapatite complex promoted bone regeneration in tibia defect model. (a-l) **Typical CT images at different time points strongly suggested that only Ihh^+^ MSCs/hydroxyapatite complex **(c, f, i, and l)** promotes bone regeneration, comparing to MSCs/hydroxyapatite complex **(a, d, g, and j)**, or EGFP^+^MSCs/hydroxyapatite complex **(b, e, h, and k)**. At 2 weeks after implantation, no obvious bone regeneration was observed in any group **(a-c)**. At 4 to 8 weeks, significantly more newly formed trabeculae were observed at the inner edges of defect sites in the Ihh^+^ EGFP^+^ MSCs/HA group **(f and i)**, comparing to control groups **(d, e, g, and h)**. At 12 weeks, the tibia defects were completely filled with new bone **(l)**, while defects persistent in control groups **(j and k)**. Note that, at the early time points (2 to 8 weeks), the cells/HA complexes can be easily identified by their unique shape and the anatomic location. In all panels, the newly generated bone could also be easily distinguished from the endogenous intact bone and cells/HA complexes by the anatomic location and variable density on the CT imaging. **(m)** The quantification of the volumes of the newly formed bone in each group (the volumes of cells/HA complexes, if there was any, were not counted as newly formed bone) **(a-l)**. Note that the volumes of the newly regenerated bone were significantly higher in the group treated with Ihh^+^ MSCs/hydroxyapatite composites from 4 weeks after implantation onward. White arrows in **(a-l)** point to injury sites. Errors bars, SD, *p< 0.05 (*n* = 5, paired *t*-test).

The complications of surgery, such as superficial or deep infection, hematoma, and bone fracture, were rarely observed in this model (only 1/40 animals had an accidental tibia fracture). Normally, within 3–4 days after surgery, rabbits showed slightly reduced appetite and activities with a little difficulty to move around. But these adversary effects were diminished at about 1 week after surgery, and no obvious intra- or inter-group behavioral difference was observed at any time point (data not shown). CT scanning confirmed that bone defect can be created in the plateau zone of all rabbit tibias consistently (Figure 2 and data not shown). The unique shape, anatomic location, and optic density on the CT imaging can also easily distinguish the newly generated bone with endogenous intact bone and the cells/HA complexes.

### Histological studies confirmed that the implanted cells survived in the long-term

**Figure 3:**
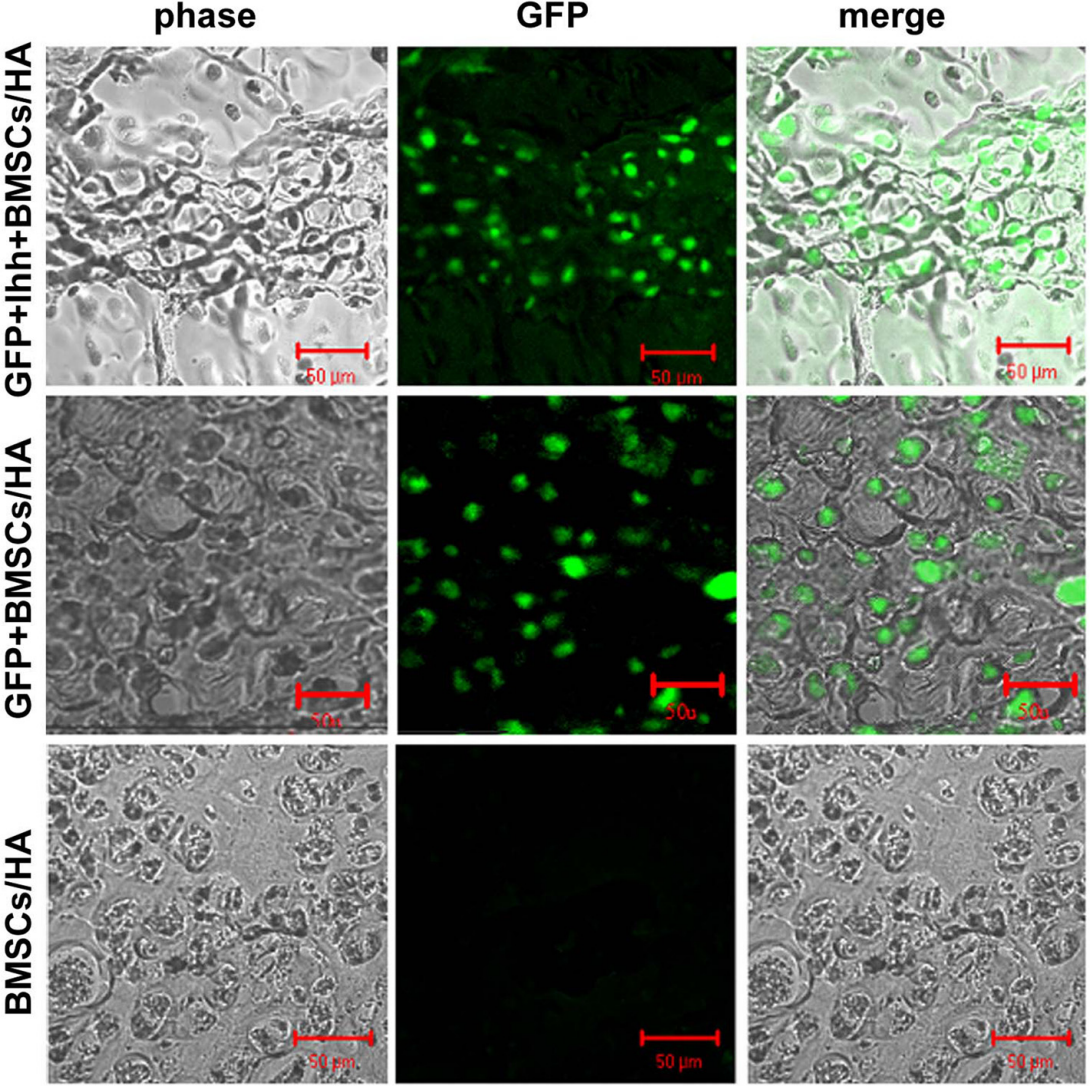
**The long-term survival of the implanted cells was confirmed by histological studies.** Histological studies were performed at different time points, i.e., 4, 8, and 12 weeks (showed) after implantation, to directly test whether EGFP^+^ were persistently present in injury site. Typical images of newly regenerated bony callus in adjacent to the endogenous bone from different groups (top row, EGFP^+^ Ihh^+^ MSCs; middle row, EGFP^+^ MSCs; bottom row, control MSCs only) are shown. Note that many EGFP^+^ cells (either with or without Ihh overexpression) were not only survived but also apparently integrated into newly generated bone in both EGFP^+^ Ihh^+^ MSCs and EGFP^+^ MSCs groups. Overall, the first column is the phase images, the second column is the florescence images of GFP, and the third column is the merged images. Scale bars = 50 μm.

To help to understand the model and interpret the final results, we implanted Ihh/MSCs/scaffold complex (EGFP^+^ Ihh^+^ MSCs/HA), in parallel with two control groups (EGFP^+^ MSCs/HA or MSCs/HA), into an established bone defect model. Histological studies at different time points were performed to directly test whether implanted cells survive in long-term *in vivo* to exert the desired effects. Interestingly, unlike some report, we found that EGFP^+^ cells were persistently present in the injury site and the newly regenerated bony callus as late as 12 weeks after implantation. More importantly, many EGFP^+^ cells in both control and case group were integrated into newly generated bone at the time (12 weeks) of most biodegradable scaffold no longer visible (Figure 3). This data must be taken into consideration into the final interpretation of the data.

### CT scanning and gross observation suggested that implanted Ihh/MSCs/scaffold complex strongly promoted bone repair

The effectiveness of implanted Ihh/MSCs/scaffold complex was evaluated first with CT scanning images. Fortunately, the newly generated bone could be easily distinguished from the endogenous intact bone and MSCs/HA complexes by the specific anatomic location and typical variable density on the CT imaging (Figure 2).

Specifically, we found that, at 2 weeks after implantation, CT scanning showed no obvious bone regeneration in any groups, and each MSCs/HA complex were clearly visible as a separate optical dense object in the burr hole.

At 4 weeks, the EGFP^+^ Ihh^+^ MSCs/HA group showed new bone formation, with mildly enhanced bone density at the inner edge, and this new bone formation was not apparent in control groups (EGFP^+^ MSCs/HA and MSCs/HA groups), and the MSCs/HA complexes were still clearly visible without obvious inter-group variation. At 8 weeks after implantation, newly formed continuous bone tissue can be observed at the defect area in EGFP^+^ Ihh^+^ MSCs/HA group, while discontinuously trabecular bone tissues were also observed in the control groups at this time point. The MSCs/HA complexes were still visible at this time point, but the optical density seemed to be decreased in all groups without obvious inter-group variation.

Interestingly, at 12 weeks, completely healed tibia defects were observed in five out of five rabbits in the EGFP^+^ Ihh^+^ MSCs/HA group (Figure 2). In contrast, this was observed in none of rabbits in control groups, even though variable degree of healing was indeed observed in both control groups. Furthermore, the unique signal of MSCs/HA complexes were almost completely disappeared at this time point (12 weeks) from all groups, which was not a surprising finding considering the biodegradability of HA complexes. Interestingly, we also noticed that the thickness of newly formed bony callus was similar to that of normal tibia (Figure 2a-l).

We quantified the volume of newly formed bone in each groups based on CT scanning, and we found an significant increase in bone regeneration in the EGFP^+^ Ihh^+^ MSCs/HA group from week 4 onward (*n* = 5, *p*< 0.05, Figure 2m). These findings strongly suggested that EGFP^+^ Ihh^+^ MSCs/HA constructs enhanced bone repair.

**Figure 4:**
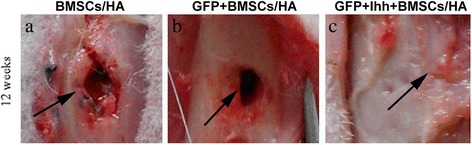
**Macroscopic observations further strengthened the idea that Ihh** ^**+**^ **MSCs/hydroxyapatite complex promote bone regeneration.** At 12 weeks after the implantation, complete bone union was observed in group implanted with EGFP^+^ Ihh^+^ MSCs/HA constructs **(c)**, while bone defects were persistent in control groups **(a and b)**. Arrows in all panels points to the injury sites. Note that the hydroxyapatite complex was undetectable in any group at this time point.

However, it is well known that CT scanning is sensitive to detect the mature bony regeneration, not the early phases of the process, such as chondrogenesis and the vasculation, thus, we reasoned that it is possible that CT scanning might fail to detect the early effects of EGFP^+^ Ihh^+^ MSCs/HA. To circumvent this limitation, we re-opened the injury sites at different time points and grossly observed the potential regeneration. At two weeks after implantation, no significant intergroup difference was found. However, unlike CT imaging, gross observation indeed detected inter-group difference as early as 4 weeks after implantation. Specifically, we found that the newly formed cartilage and well-arranged capillary network were visible in the injuries sites and surrounding area in the EGFP^+^ Ihh^+^ MSCs/HA group but not in control groups (Additional file 4: Figure S4). More importantly, similar to CT scanning, this inter-group difference was persistent, i.e., complete healing was only observed in EGFP^+^ Ihh^+^ MSCs/HA group, at 12 weeks after implantation not in control groups (Figure 4).

Overall, as expected, the only obvious difference is that the gross observation is more sensitive to detect the changes in the early healing process. Together, these observations suggested that implanted Ihh/cell/scaffold complex promoted bone repair. However, the observed relatively late effects raised the possibility that Ihh/MSCs/scaffold complex worked through indirect mechanisms, even though gross observation suggested that enhanced chondrogenesis, osteogenesis, and angiogenesis/vasculation might play key roles in this process.

### Enhanced osteogenesis and angiogenesis in EGFP^+^ Ihh^+^ MSCs/HA implanted group

**Figure 5:**
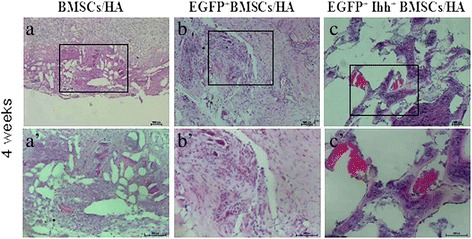
**Histological study found enhanced osteogenesis and angiogenesis in Ihh** ^**+**^ **MSCs/hydroxyapatite group.** Typical images at 4 weeks after implantation of MSCs/hydroxyapatite complex **(a and a′)**, EGFP^+^ MSCs/hydroxyapatite complex **(b and b′)**, and EGFP^+^Ihh^+^ MSCs/hydroxyapatite complex **(c and c′)** show the histological features adjacent to the implantation sites in different groups. Low power images are shown in **(a)**, **(b)**, and **(c)**, and **(a′)**, **(b′)**, **(c′)** are the higher power images of boxed area from **(a)**, **(b)**, and **(c)**, respectively. Note that only **(c)** and **(c′)** shows the typical histological features of newly formed trabeculae and nascent angiogenesis. Scale bars = 100 μm.

We next performed histological study to further understand how EGFP^+^ Ihh^+^ MSCs/HA implants promote bone repair in this model. Specifically, at 4 weeks following implantation, histological study found that there were many cells inside the microchannels and around the HA scaffolds in all three groups. Even though the total cell density seemed not significantly different among three different groups, obvious bony callus were only found around the implants in EGFP^+^ Ihh^+^ MSCs/HA group, suggesting enhanced osteogenesis in this group (Figure 5). Similar trend was also observed in later time points (data not shown). Together, histological study, gross observation, and CT scanning all suggested the increased chondrogenesis, osteogenesis, and angiogenesis only in EGFP^+^ Ihh^+^ MSCs/HA group.

However, due to the long course of healing and the complicate *in vivo* microenvironment, we reasoned that it is beyond the scope of this study to fully delineate the exact sequence of the events *in vivo*. Furthermore, lack of high affinity rabbit lineage specific antibodies also limited us to directly observe the potential osteogenic, chondrogenic, or angiogenic fate changes of the donor cells *in vivo*.

### *In vitro* study not only strengthened the idea that high Ihh signaling could promote chondrogenesis, angiogenesis, and osteogenesis but also revealed potential feedback Ihh signaling regulation

**Figure 6:**
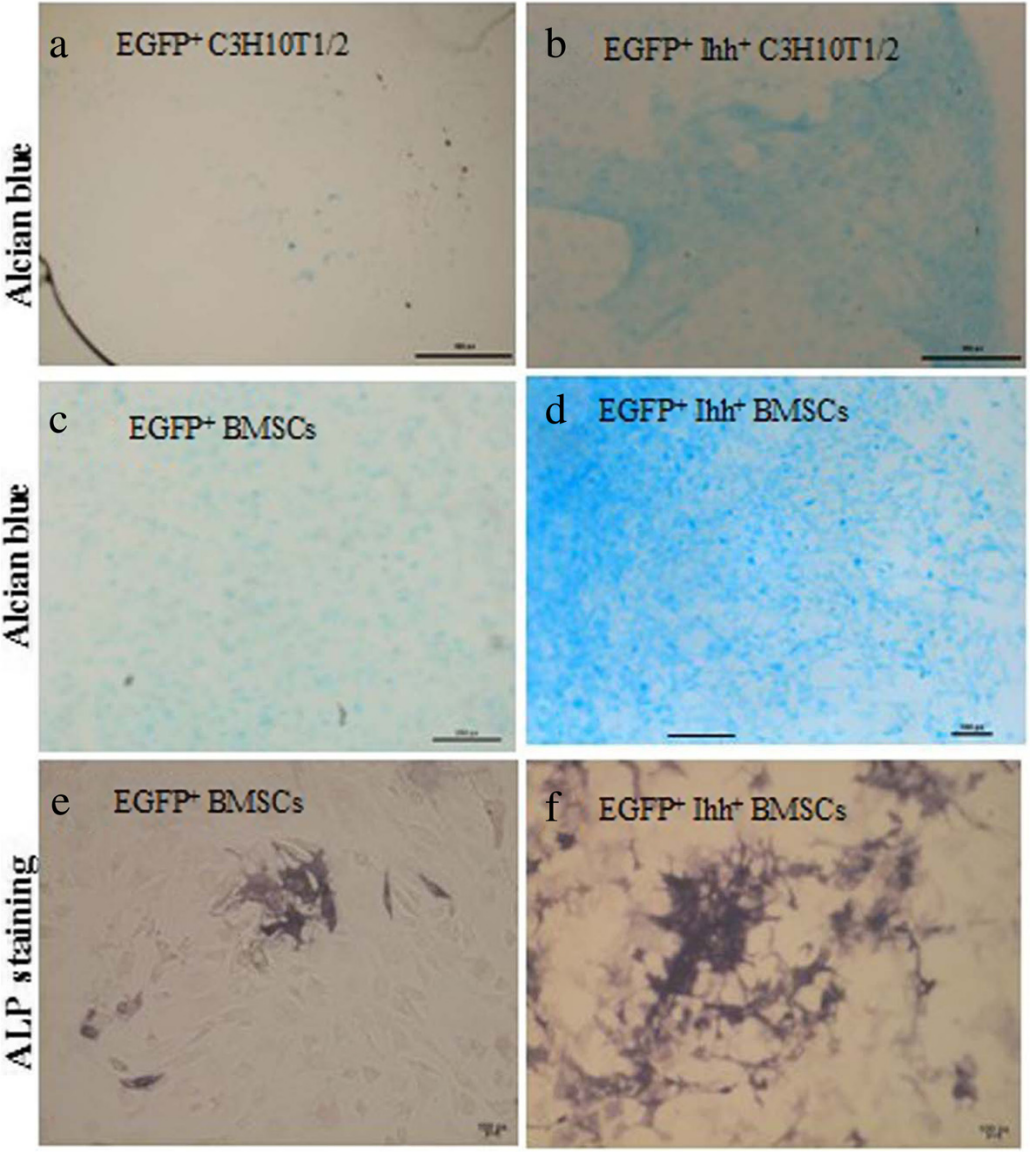
**Ihh promotes the expression of chondrogenic and osteogenic markers** ***in vitro*** **.** Both C3H10T1/2 cells **(a and b)** and rabbit MSCs **(c-f)** were used in this study. Typical images demonstrated that under chondrogenic condition **(a-d)**, strong Alcian Blue staining was observed in both EGFP^+^ Ihh^+^ C3H10T1/2 cells **(b)** and EGFP^+^ Ihh^+^ MSCs **(d)** 21 days after transfection, while only minimum Alcian Blue staining was observed in control groups **(a and c)**. Similarly, under osteogenic condition **(e and f)**, strong ALP^+^ staining was observed only in EGFP^+^ Ihh^+^ MSCs **(f)** not in MSCs group **(e)**, 7 days after transfection. Scale bars = 100 μm.

For abovementioned reasons, we next turned our attention to more controlled *in vitro* experiments to further understand the underlying cellular and molecular mechanisms. Consistent with the *in vivo* studies, we found that overexpression of Ihh in C3H10T1/2 or MSCs cells led to high level of Alcian Blue staining (Figure 6a–d). ALP staining also demonstrated that Ihh induced an elevated ALP expression in MSCs (Figure 6e–f). Consistently, *in vitro* IHC study also found that Ihh-transfected C3H10T1/2 cells showed upregulated expression of osteocalcin (Oc, a marker of the mature osteoblasts) and CD31 (an endothelial cell marker), 21 days after gene transfection (Additional file 5: Figure S5). More interestingly, higher expression of CD31 or Oc is not consistently co-localized with GFP, which might suggest that Ihh signaling could work through non-cell-autonomous mechanisms.

**Figure 7:**
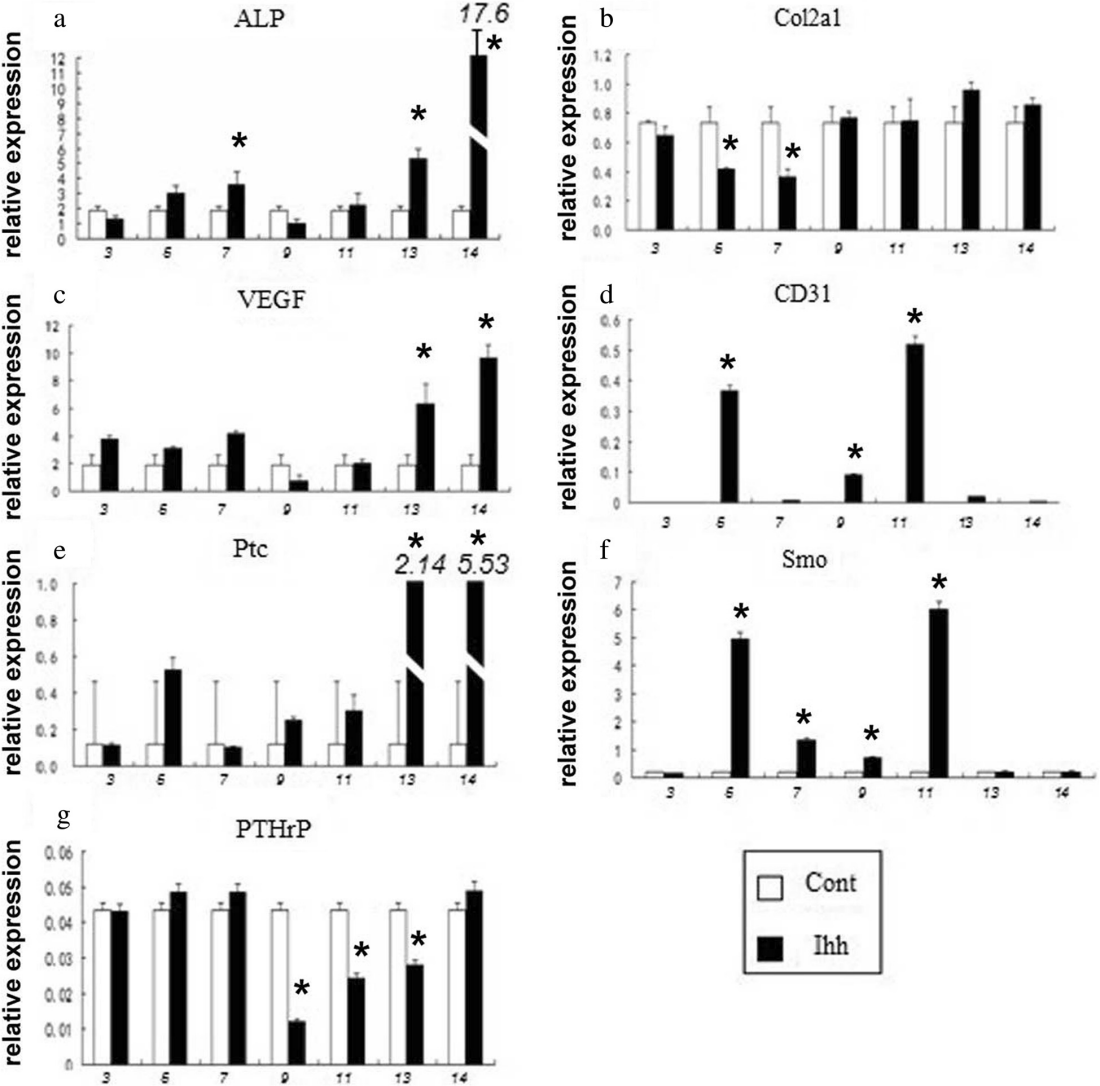
**Ihh differentially regulates the expression of markers of osteogenic, chondrogenic, angiogenic, and Ihh signaling genes.** Consistent with the previous study, the expression of the earliest osteogenic marker gene, ALP, was almost always upregulated **(a)**. Similarly, angiogenic factor (VEGF) and markers (CD31) were also mostly upregulated **(c and d)**. Strangely, for unknown reasons, Col2a1 a chondrogenic marker was only marginally regulated by Ihh in C3H10T1/2 cells **(b)**. Ptc1 and Smo, Ihh receptors, are upregulated by Ihh at most time points **(e and f)**. In contrast, the expression of PTHrP, a factor in the negative feedback loop of Ihh signaling, was unchanged or suppressed at some time points in C3H10T1/2 cells **(g)**. *p< 0.05, statistical significance (paired *t*-test, comparing with control group at the same time point).

In addition, RT-qPCR (Figure 7) and RT-PCR (Additional file 6: Figure S6) were used to further probe the underlying molecular mechanisms and the Ihh signaling regulation. By RT-qPCR, we found that Ihh not only induced the upregulation of molecular markers of angiogenic factor (VEGF), angiogenic marker (CD31), and osteogenesis (ALP) but also induced factors that might potentially feedback regulate the Ihh signaling regulation (Ptc1, Smo, and PTHrP) in C3H10T1/2 cells (Figure 7). The general regulated expression patterns were repeated in another set of RT-PCR (Additional file 6: Figure S6).

## Discussion

Numerous previous reports indicated that Ihh signaling not only controls chondrocyte and osteoblast development but also promotes cartilage vascularization [14,15], in the normal physiological process of endochondral bone formation. For example, Vortkamp lab [24] has shown that Ihh and PTHrP are secreted by prehypertrophic chondrocytes and then have an effect on neighboring or distant cells (through non-cell autonomous mechanisms). The feedback loop of Ihh/PTHrP co-regulates the proper development of cartilage tissue and promote vascular invasion in the prehypertrophic stage [25], at least partially through synchronizing and determining the pace of differentiation of chondrocytes and osteoblasts in the growth plate [9,14].

In acute injury context, previous data also indicate that Ihh is expressed in chondrocytes and osteoblasts during the process of fracture healing in adult rat femora [26], consistently, the activation of hedgehog signaling during fracture repair enhances osteoblastic-dependent matrix formation [13]. These data all suggested that Ihh might also play a role in the regulation of bone repair or remodeling in adult animals. Additional study in the regenerating deer antler further suggested that molecules which regulate embryonic skeletal development and postnatal epiphyseal growth, including Ihh, may also control blastema formation, chondrogenesis, and bone formation. However, no investigator has specifically tested whether and how high Ihh signaling could regulate adult bone regeneration after acute injury in a clinically relevant model.

To the best of our knowledge, this is the first study that directly tested the functional consequences of local application of high Ihh signaling through MSCs in a clinically relevant context. In this proof-of-concept study, we generated MSCs/scaffold complex using MSCs transfected with EGFP^+^ Ihh^+^ viruses and implant this construct into an *in vivo* model of rabbit tibia defect. Our results of CT imaging and gross and histological observations revealed that implanted EGFP^+^ Ihh^+^ MSCs/HA constructs significantly accelerated the bone regeneration. This finding indicated that high Ihh signaling could promote adult bone regeneration after acute injury. This conclusion has been well supported by our data and is of great interest.

However, to extensively delineate the exact sequence of the cellular or molecular events or prove the causal relationship *in vivo* or *in vitro* is far beyond the scope of this study, nevertheless, our current *in vitro* and *in vivo* studies did suggest that enhanced chondrogenesis, osteogenesis, and angiogenesis might be all involved in the relatively long healing process. Our double-staining and other data further suggested that Ihh could work through non-cell-autonomous mechanism, however, the possibility of cell-autonomous effects can not completely excluded by our current data. For this reason, more detailed future studies will be needed to further clarifying the underlying molecular mechanisms.

It is worthy to mention that even though it is known that endogenous mesenchymal stem/progenitor cells contribute to the maintenance of healthy tissues or as immunomodulatory sentinels, and MSCs from different sources with a variety of modifications have also been widely applied in regenerative medicines in different clinical contexts [18,19], control MSCs without Ihh overexpression were far less efficient in promoting the bone healing process, comparing to MSCs with Ihh overexpression in our study.

An obvious limitation of our current study is that this study did not have a sham surgery group that did not receive the MSCs. For this reason, we could not tell for sure whether MSCs alone have any pro-healing activity. Another limitation is that, in our *in vitro* study, unlike the commonly accepted *in vivo* finding, overexpression of Ihh in C3H10T1/2 cells was unable to induce the PTHrP gene (Figure 7), which may reflect the absence of specific microenvironmental factors *in vitro* [27], alternatively, the induction of PTHrP was failed to be detected simply because the induction was transient or happened only in an insignificant small subpopulation. It is also interesting to notice that the mRNA levels of BMP2 and BMP4 were not significantly changed in our *in vitro* study either, even though Duench and Franz-Odendaal reported that BMPs might be direct targets of Ihh signaling [28]. Other limitations of our current studies are the following: 1) the size of rabbit tibial plateau precluded the possibility to establish a bigger defect, which might be more sensitive in detecting the pro-healing effects of implant,and 2) the safety issue of retroviral system excluded the possibility of direct clinical application of our system.

Nevertheless, the implications of this study are at least twofold: for the basic study of Ihh signaling, we reason that this study may provide a valuable framework and foundation to further delineate the potential underlying molecular and cellular mechanisms, and this working model may be even useful for studying the crosstalking of Ihh signaling with other key signaling pathways such as Wnts, BMPs, and other growth factors. For the clinical front, the similar approach could potentially apply to clinical treatment of acute bone defect, if the safety concern of the viral delivery system has been appropriately addressed.

## Conclusions

In summary, we found that implanted Ihh^+^ MSCs/hydroxyapatite enhanced bone regeneration effectively in a clinically relevant acute injury model. Even though the exact underlying mechanisms are still far from clear, our primary data suggested that enhanced chondrogenesis, osteogenesis, and angiogenesis of mesenchymal stem/progenitor cells could at least partially contribute to the healing process. This study not only has implications for basic research of Ihh signaling pathway but also points to the possibility of direct application of this specific paradigm to clinical bone repair.

## Additional files

Additional file 1: Figure S1. Map and the evaluation of Ihh overexpression plasmid. **(A)** Map of pSFG-EGFP-Ihh. **(B)** C3H10T1/2 cells infected with retrovirus expressed GFP. **(C)** Expression of Ihh protein was evaluated by Western blotting. By staining with flag antibody, a specific 46 kDa band was detected only in Ihh transfected C3H10T1/2 cells. Cont, C3H10T1/2 cells transfected with EGFP gene; Ihh, C3H10T1/2 cells transfected with EGFP and Ihh genes. Scale bars = 250 μm. Additional file 2: Figure S2. Schematic views of implantation of cells/HA complex into bone defects. **(a)** Rabbit tibia defect model. **(b)**Schematic view of the size of HA scaffold and burr hole in rabbit tibia defect model. Note that HA scaffold was well-fitted in the burr hole. Additional file 3: Figure S3. Observation of EGFP^+^Ihh^+^MSCs inside HA complex. **(A)** Tightly arrayed GFP^+^ cells were observed inside HA complex under inverted fluorescence microscope. **(B) **Representative SEM picture showed that the typical spindle-like shape cells were attached to HA scaffolds. **(A)** and **(B)** essentially confirmed that the seeded cells survived on the surface of HA scaffolds. Scale bars = 100 μm in **(A)**. Scale bars = 50 μm in **(B)**. Additional file 4: Figure S4. Gross observations found newly formed cartilage and well-organized capillary network four weeks after implantation in the EGFP^+^Ihh^+^MSCs/HA group only. Boxed areas **(a)**, **(b)**, and **(c)** are shown at higher magnification in **(a′)**, **(b′)**, and **(c′)**, respectively. Implants were removed from **(a′)**, **(b′)**, and **(c′)** to show the inner side of defect sites. New cartilage tissues were observed in the inner side of defect site (**(c′)**, black arrows indicate cartilage tissues). Well-arranged capillary network showing in figure **(c′)** (white arrows indicate capillaries). Additional file 5: Figure S5. Ihh induced CD31 and Oc expression in C3H10T1/2 cells. **(a)** Typical images suggested induction of CD31 (red, bottom panel) by overexpression of Ihh (GFP^+^) comparing with none transfected cells (GFP^−^, green, upper panel), 21 days after gene transfection. **(b)** Typical images also demonstrated overexpression of Ihh (GFP^+^) upregulated Oc (red, bottom panels), comparing with none transfected cells (GFP^−^, green, upper panel), 21 days after gene transfection. Blue in all panels is DAPI stain. Ihh, EGFP^+^ Ihh^+^ C3H10T1/2 cells; Cont, C3H10T1/2 cells. In both case groups (CD31 and Oc), the upregulation was not limited to GFP^+^ cells, which suggested that the underlying mechanisms could be non-cell autonomous. Scale bars = 50 μm. Additional file 6: Figure S6. RT-PCR also found that overexpression of Ihh significantly regulated many important differentiation related genes in C3H10T1/2 cells. Typical gel images showed the gene expression patterns of differentiation-related genes 14 (14d) and 21 (21d) days after gene transfection. β-actin served as internal control. Control (cont), C3H10T1/2 cells transfected with EGFP gene. Ihh, C3H10T1/2 cells transfected with EGFP and Ihh genes. H_2_O, negative control. Factors of the hedgehog pathway (Ptc1, Smo, and Gli1~3), osteogenic or chondrocyte markers (ColX, ColI, Oc, Aggrecan), vascular cell markers (VEGF, Flk-1, CD31, aSMA), BMPs, and BMP target (BMP2 and 4, RUNX2) were tested. Note that many genes are differentially regulated but generally consistent with previous RT-qPCR data.
