# Supplementary figures and images for: Mesenchymal stem cells overexpressing Ihh promote bone repair
Source: J Orthop Surg Res. 2014 Oct 28;9:102. doi: 10.1186/s13018-014-0102-7 (PMC4213494; doi:10.1186/s13018-014-0102-7)

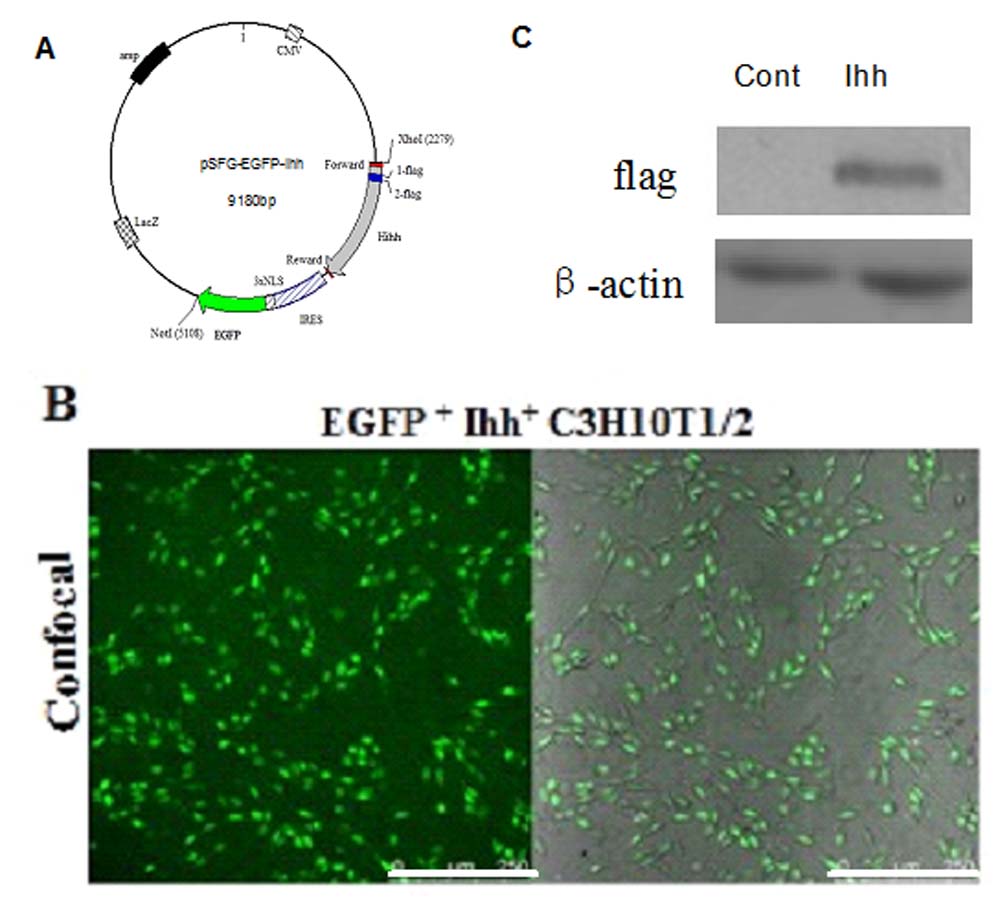

Supplement: Additional file 1: Figure S1. — Map and the evaluation of Ihh overexpression plasmid. (A) Map of pSFG-EGFP-Ihh. (B) C3H10T1/2 cells infected with retrovirus expressed GFP. (C) Expression of Ihh protein was evaluated by Western blotting. By staining with flag antibody, a specific 46 kDa band was detected only in Ihh transfected C3H10T1/2 cells. Cont, C3H10T1/2 cells transfected with EGFP gene; Ihh, C3H10T1/2 cells transfected with EGFP and Ihh genes. Scale bars = 250 μm. [file 13018_2014_102_MOESM1_ESM.jpeg]

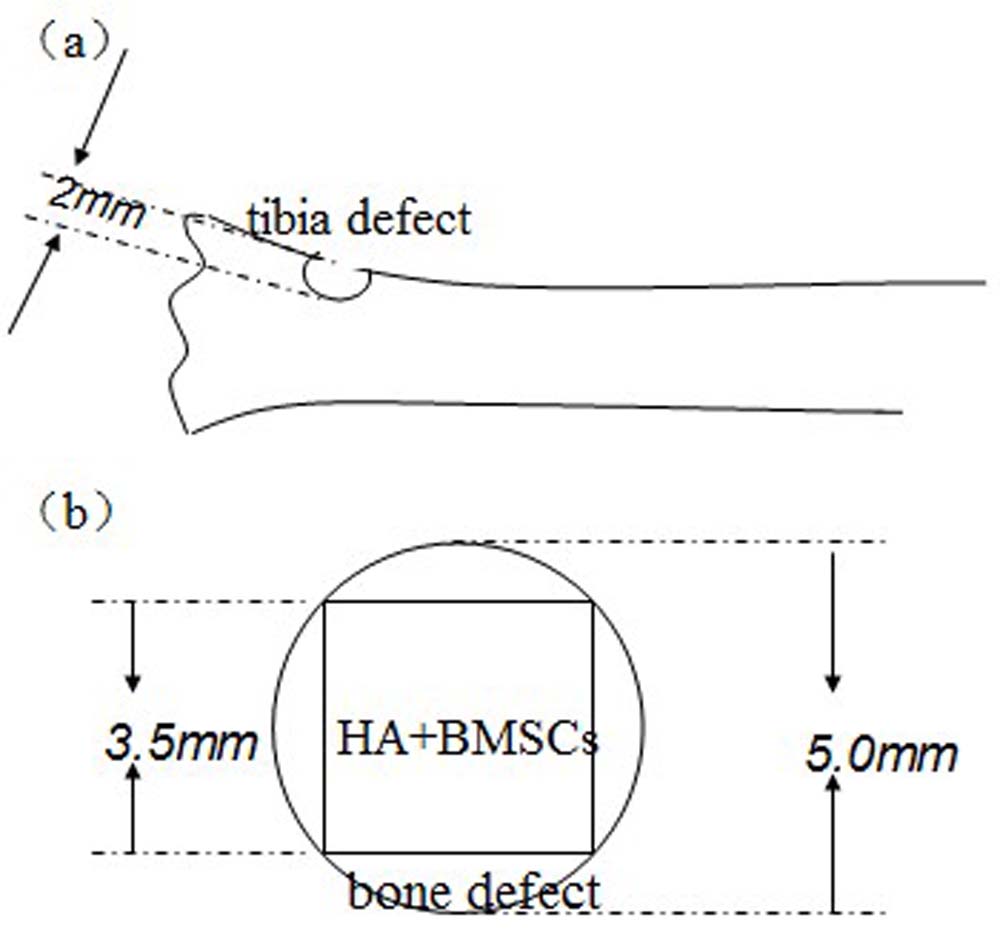

Supplement: Additional file 2: Figure S2. — Schematic views of implantation of cells/HA complex into bone defects. (a) Rabbit tibia defect model. (b)Schematic view of the size of HA scaffold and burr hole in rabbit tibia defect model. Note that HA scaffold was well-fitted in the burr hole. [file 13018_2014_102_MOESM2_ESM.jpeg]

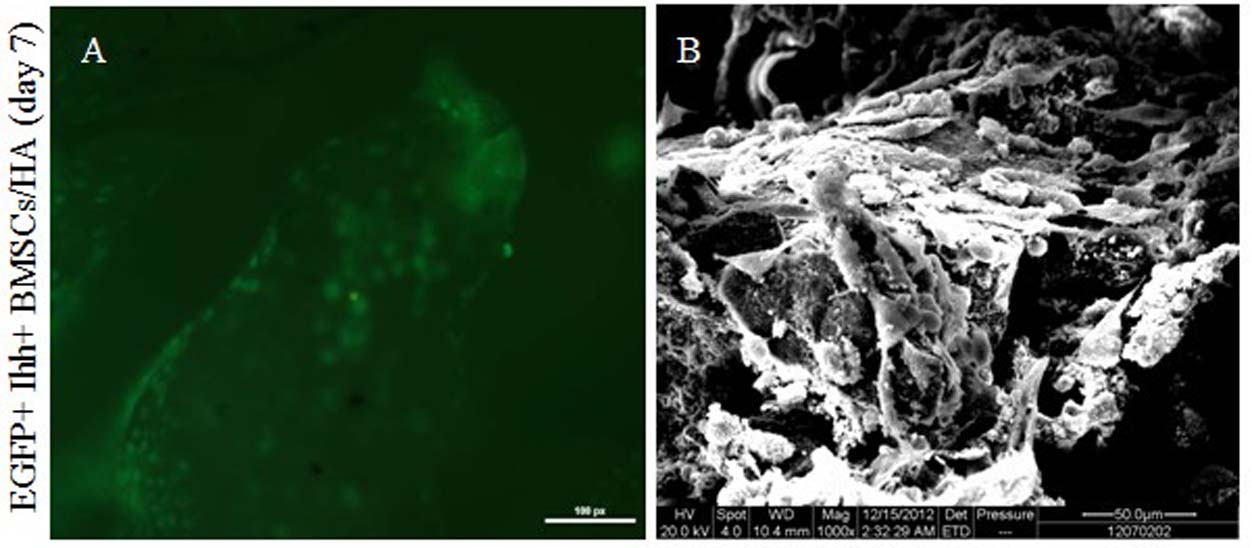

Supplement: Additional file 3: Figure S3. — Observation of EGFP+Ihh+MSCs inside HA complex. (A) Tightly arrayed GFP+ cells were observed inside HA complex under inverted fluorescence microscope. (B) Representative SEM picture showed that the typical spindle-like shape cells were attached to HA scaffolds. (A) and (B) essentially confirmed that the seeded cells survived on the surface of HA scaffolds. Scale bars = 100 μm in (A). Scale bars = 50 μm in (B). [file 13018_2014_102_MOESM3_ESM.jpeg]

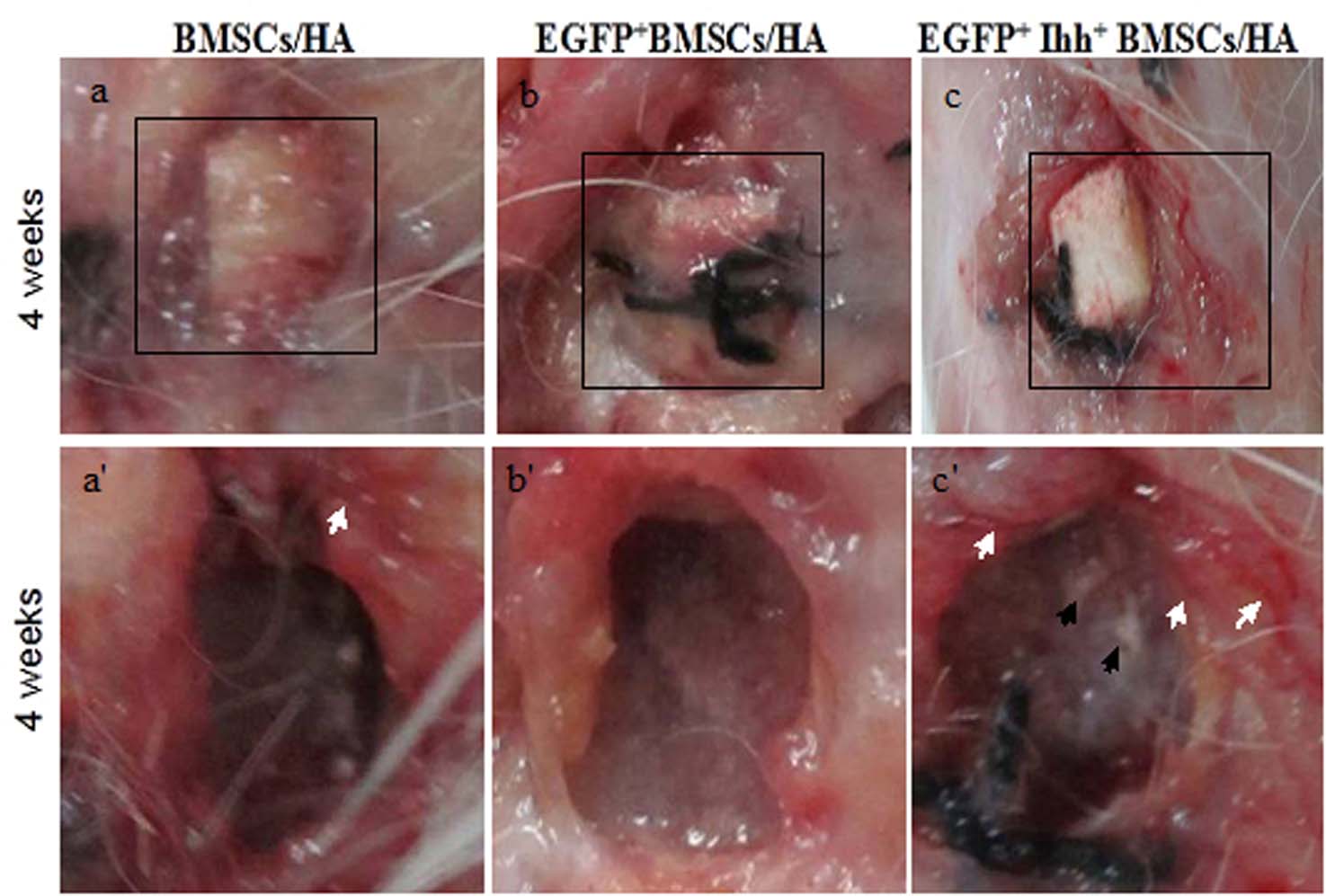

Supplement: Additional file 4: Figure S4. — Gross observations found newly formed cartilage and well-organized capillary network four weeks after implantation in the EGFP+Ihh+MSCs/HA group only. Boxed areas (a), (b), and (c) are shown at higher magnification in (a′), (b′), and (c′), respectively. Implants were removed from (a′), (b′), and (c′) to show the inner side of defect sites. New cartilage tissues were observed in the inner side of defect site ((c′), black arrows indicate cartilage tissues). Well-arranged capillary network showing in figure (c′) (white arrows indicate capillaries). [file 13018_2014_102_MOESM4_ESM.jpeg]

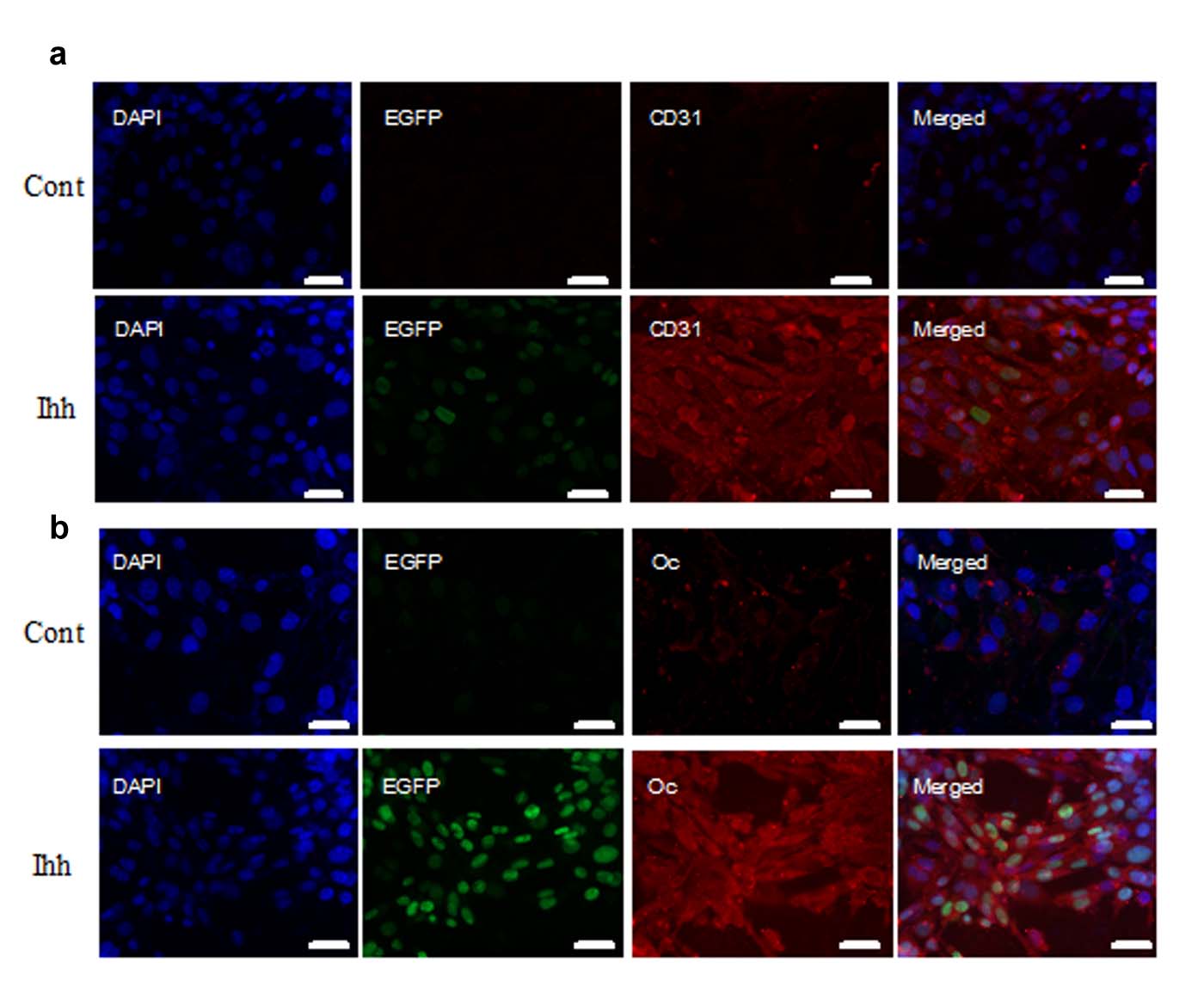

Supplement: Additional file 5: Figure S5. — Ihh induced CD31 and Oc expression in C3H10T1/2 cells. (a) Typical images suggested induction of CD31 (red, bottom panel) by overexpression of Ihh (GFP+) comparing with none transfected cells (GFP−, green, upper panel), 21 days after gene transfection. (b) Typical images also demonstrated overexpression of Ihh (GFP+) upregulated Oc (red, bottom panels), comparing with none transfected cells (GFP−, green, upper panel), 21 days after gene transfection. Blue in all panels is DAPI stain. Ihh, EGFP+ Ihh+ C3H10T1/2 cells; Cont, C3H10T1/2 cells. In both case groups (CD31 and Oc), the upregulation was not limited to GFP+ cells, which suggested that the underlying mechanisms could be non-cell autonomous. Scale bars = 50 μm. [file 13018_2014_102_MOESM5_ESM.jpeg]

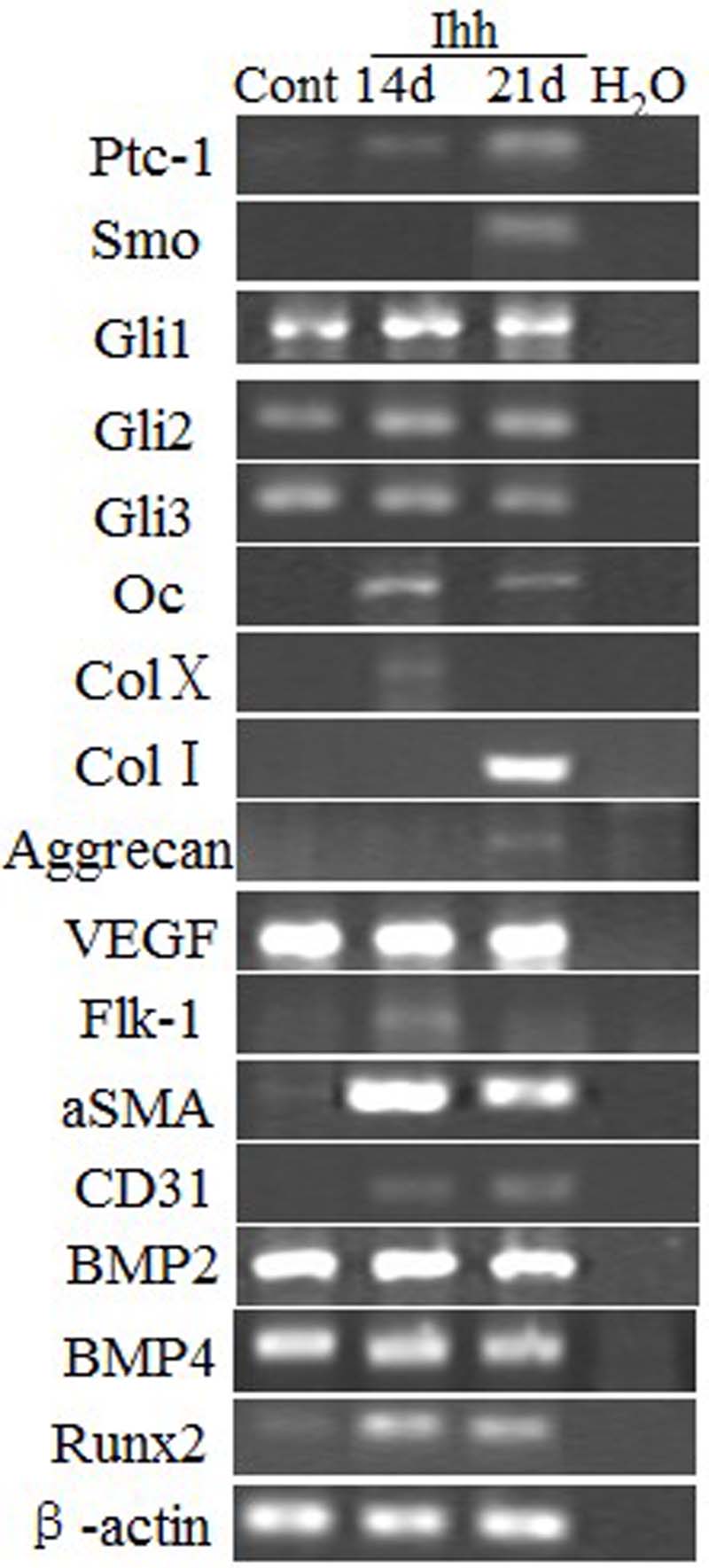

Supplement: Additional file 6: Figure S6. — RT-PCR also found that overexpression of Ihh significantly regulated many important differentiation related genes in C3H10T1/2 cells. Typical gel images showed the gene expression patterns of differentiation-related genes 14 (14d) and 21 (21d) days after gene transfection. β-actin served as internal control. Control (cont), C3H10T1/2 cells transfected with EGFP gene. Ihh, C3H10T1/2 cells transfected with EGFP and Ihh genes. H2O, negative control. Factors of the hedgehog pathway (Ptc1, Smo, and Gli1~3), osteogenic or chondrocyte markers (ColX, ColI, Oc, Aggrecan), vascular cell markers (VEGF, Flk-1, CD31, aSMA), BMPs, and BMP target (BMP2 and 4, RUNX2) were tested. Note that many genes are differentially regulated but generally consistent with previous RT-qPCR data. [file 13018_2014_102_MOESM6_ESM.jpeg]
